# The Shift in Key Functional Traits Caused by Precipitation under Nitrogen and Phosphorus Deposition Drives Biomass Change in *Leymus chinensis*

**DOI:** 10.3390/plants12091781

**Published:** 2023-04-26

**Authors:** Ruqiang Tong, Xinran Yang, Qiuyue Wang, Lin Li, Yanan Li, Yujie Shi, Chunsheng Mu, Junfeng Wang

**Affiliations:** 1 Key Laboratory of Vegetation Ecology of the Ministry of Education, Institute of Grassland Science, Jilin Songnen Grassland Ecosystem National Observation and Research Station, Northeast Normal University, Changchun 130024, China; tongrq687@nenu.edu.cn (R.T.); yangxr157@nenu.edu.cn (X.Y.); lliyn903@nenu.edu.cn (Y.L.); shiyj455@nenu.edu.cn (Y.S.); mucs821@nenu.edu.cn (C.M.); 2 School of Life Sciences, Northeast Normal University, Changchun 130024, China; wangqy582@nenu.edu.cn; 3 Jilin Agricultural Radio and Television School, Changchun 130599, China

**Keywords:** biomass, coupling effect, *Leymus chinensis*, nitrogen or phosphorus deposition, precipitation change, shift in functional traits

## Abstract

The trade-offs between key functional traits in plants have a decisive impact on biomass production. However, how precipitation and nutrient deposition affect the trade-offs in traits and, ultimately, productivity is still unclear. In the present study, a mesocosm experiment was conducted to explore the relationships between biomass production and the aboveground and belowground key functional traits and their trade-offs under changes in precipitation and nutrient depositions in *Leymus chinensis*, a monodominant perennial rhizome grass widespread in the eastern Eurasian steppe. Our results showed that moisture is the key factor regulating the effect of nitrogen (N) and phosphorus (P) deposition on increased biomass production. Under conditions of average precipitation, water use efficiency (WUE) was the key trait determining the biomass of *L. chinensis*. There were obvious trade-offs between WUE and leaf area, specific leaf area, leaf thickness, and leaf dry matter. Conversely, under increasing precipitation, the effect of restricted soil water on leaf traits was relieved; the key limiting trait changed from WUE to plant height. These findings indicate that the shift of fundamental traits of photosynthetic carbon gain induced by precipitation under N and P deposition is the key ecological driving mechanism for the biomass production of typical dominant species in semi-arid grassland.

## 1. Introduction

Generally, primary productivity in grassland ecosystems is strongly driven by soil environmental factors such as precipitation and nitrogen [1]. Any subtle changes in soil moisture and nutrient availability can have a significant impact on productivity [2]. Studies have shown that when nutrients are added (such as N), the productivity of dry grassland will increase significantly with an increase in precipitation [3,4]. This is related to the change in species abundance caused by the coupling effect between water and nutrients such as N [5]. When productivity fluctuations at the community level are reflected at the population level, they are manifested as changes in species functional traits, especially in the case of leaf functional traits related to photosynthesis [6], such as leaf area, specific leaf area, leaf dry matter content, and chlorophyll content [7]. Therefore, the relationship between environmental changes and plant functional traits can objectively reflect the response of plants to environmental changes, which has important theoretical guiding significance for the adaptive management of grassland in the future.

Changes in precipitation patterns, atmospheric dry and wet N deposition, and dry P deposition, as well as their impacts on the function of the ecosystem in which these changes occur, are among the hottest topics in ecology [8]. When the environment is water-limited, plants will improve leaf moisture-holding capacity by significantly reducing leaf area and specific leaf area and increasing leaf dry matter content. Simultaneously, plants could adapt to water stress by increasing leaf nutrient content to enhance drought resistance [9]. Generally, plant biomass increases with the increase in moisture content, whereas excessive soil moisture inhibits plant growth [10]. As limiting elements for plant growth and development, N and P are functionally coupled in ecosystems [11,12]. The effects of N and P are closely related to soil moisture, and the interaction between water, N, and P can significantly affect plant functional traits. Under the conditions of N and P addition, the increase in precipitation augmented the leaf area, photosynthetic rate, and aboveground biomass, whereas the specific leaf area decreased with the increase in precipitation. When soil moisture and nutrients change simultaneously, plants can balance different functional traits and rapidly adapt leaf traits through phenotypic plasticity to maximize their fitness for growth and development [13,14]. In general, the interactive effect of moisture and nutrients can affect the growth, development, and productivity of plants. Nevertheless, in the case of increased availability of N and P, it remains unclear how changes in precipitation influence productivity by affecting the trade-off between key functional traits. The objective of this study was to investigate the effects of N and P deposition on the key functional traits that are related to photosynthetic carbon capture and biomass production in perennial grass. Our study can provide a reference for predicting the response of grassland ecosystems to global changes.

*Leymus chinensis* is a typical perennial rhizome grass that is distributed in the meadow steppe at the eastern end of the Eurasian steppe zone, with biomass accounting for 80–90% of the community biomass [4,15]. It has the characteristics of fast growth, salt–alkali tolerance, large biomass, good palatability, high nutritional value, and strong vegetative reproduction ability. Therefore, we used this species to explore the effects of N and P deposition on biomass production under different precipitation conditions by analyzing the changes in key functional traits, which will be helpful for better understanding the changes in grassland productivity in semi-arid areas. We predicted that (1) precipitation would significantly affect the yield of *L. chinensis*, and the yield would increase with increases in precipitation within a certain range; (2) under average precipitation conditions, the effects of N and P deposition on the biomass would be related to key characteristics affecting water absorption and utilization; under increased precipitation, the N and P deposition would be related to plant height due to the easing of water restrictions; and (3) the trade-offs between key functional traits related to photosynthesis would be the main drivers of biomass changes under changes in precipitation and nutrients.

## 2. Results

### 2.1. Biomass and Its Components

**Figure 1:**
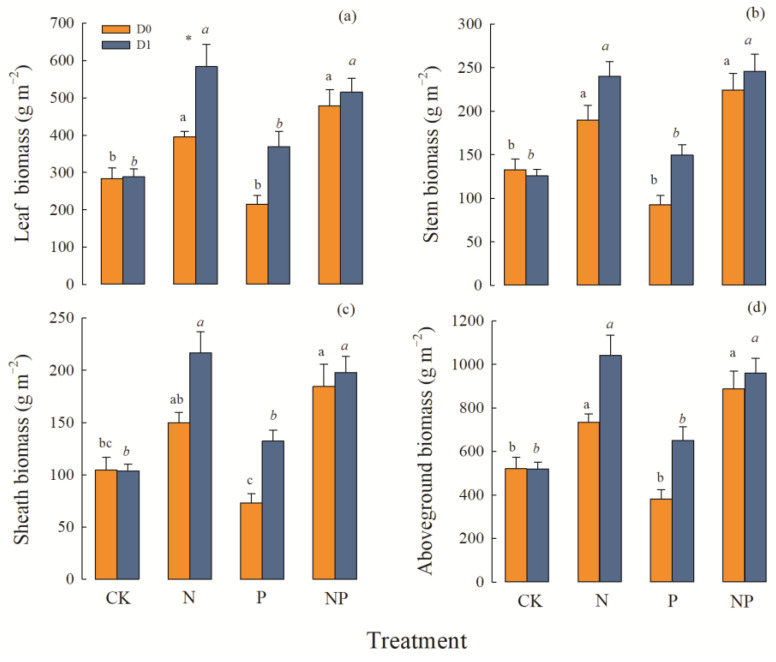
Effects of precipitation and nutrient deposition on the leaves, stems, sheaths, and aboveground biomass of *L. chinensis* (mean ± SE, *n* = 6). CK—control; N—simulated nitrogen deposition; P—simulated phosphorus deposition; NP—N and P co-deposition; D0—average precipitation; D1—increased precipitation. Different lowercase letters indicate significant differences (*p* < 0.05) among different nutrient-addition treatments under the same precipitation condition. The asterisk indicate significant differences (* *p* ≤ 0.05) among different precipitation condition under the same nutrient-addition treatments. (**a**) Effects of precipitation and nutrient deposition on the leaves biomass of *L. chinensis*. (**b**) Effects of precipitation and nutrient deposition on the stems biomass of *L. chinensis*. (**c**) Effects of precipitation and nutrient deposition on the sheaths biomass of *L. chinensis*. (**d**) Effects of precipitation and nutrient deposition on the aboveground biomass of *L. chinensis*.

The main factors of precipitation and N addition, as well as the precipitation × N × P interactions, all had significant effects on the leaf, stem, sheath, and aboveground biomass in *L. chinensis* (Table 1). Under conditions of both average precipitation and increased precipitation, the simulated N deposition and the N and P co-deposition significantly increased the leaf, stem, sheath, and aboveground biomass, and the effects of the two treatments on the total biomass and each component factor showed the same changes. In comparison to the control, N deposition and N and P co-deposition separately increased total biomass by 40% and 69%, respectively, under average precipitation conditions and increased it by 106% and 82% under increased precipitation. Among all treatment combinations, the leaf, sheath, and aboveground biomass reached their largest values with the single N deposition treatment under increasing precipitation, and the aboveground biomass was up to 1040.55 g m^−2^ (Figure 1d). Under average precipitation, P deposition had no significant effect on biomass, whereas P deposition resulted in a non-significant promoting effect on biomass production under increased precipitation. However, this effect was obviously weaker than under N deposition and N and P co-deposition.

### 2.2. Functional Traits of Individuals

#### 2.2.1. Plant Height and Leaf Morphology Traits

**Figure 2:**
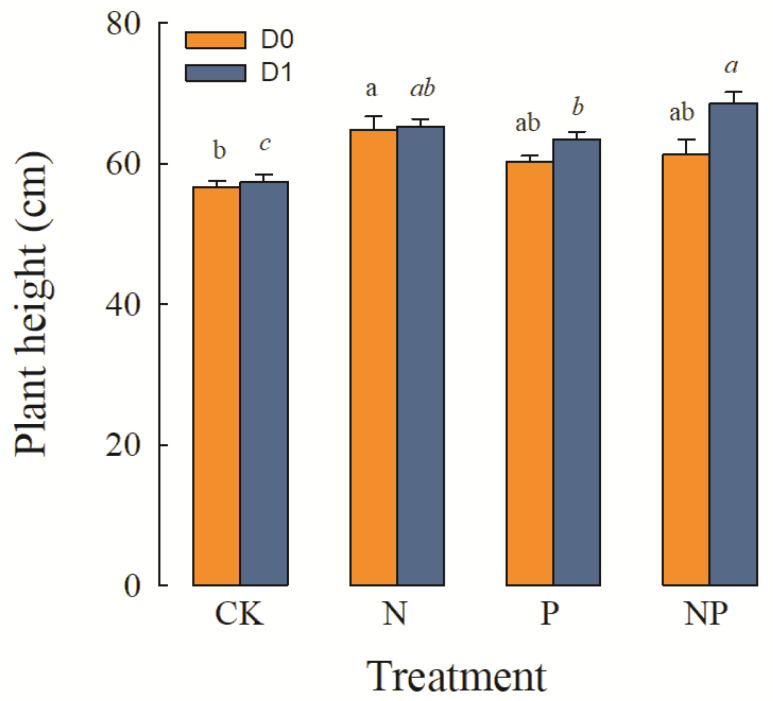
Effects of precipitation and nutrient deposition on the plant height of *L. chinensis* (mean ± SE, *n* = 6). CK—control; N—simulated nitrogen deposition; P—simulated phosphorus deposition; NP—N and P co-deposition; D0—average precipitation; D1—increased precipitation. Different lowercase letters indicate significant differences (*p* < 0.05) among different nutrient-addition treatments under the same precipitation condition.

Plant height was significantly affected by precipitation, the simulated N deposition and P deposition main factors, and the precipitation × P and the N × P interactions (Table 1). Under average precipitation, the plant height under simulated N deposition was the greatest, being 7.56%, 5.71% greater than under P deposition and N and P co-deposition, respectively. When precipitation was increased, N deposition, P deposition, and N and P co-deposition significantly increased the plant height, with plant height under N and P co-precipitation reaching its highest level (Figure 2).

**Figure 3:**
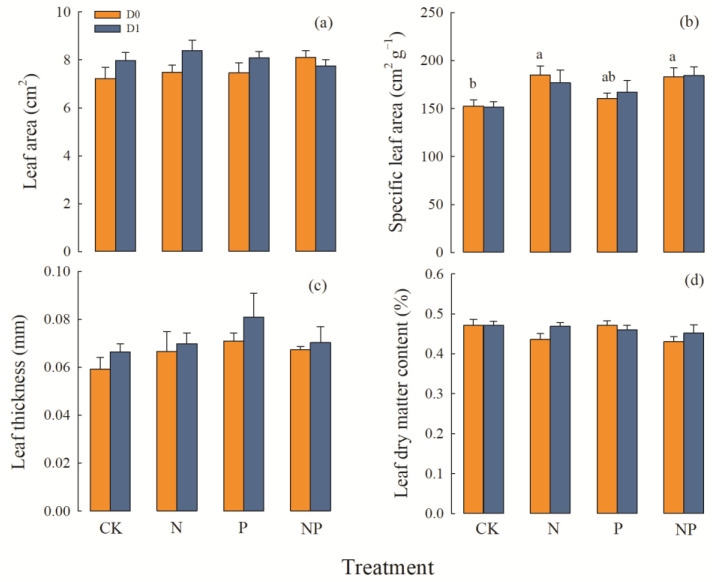
Effects of precipitation and nutrient deposition on the leaf morphology functional traits of *L. chinensis* leaves (mean ± SE, *n* = 6). CK—control; N—simulated nitrogen deposition; P—simulated phosphorus deposition; NP—N and P co-deposition; D0—average precipitation; D1—increased precipitation. Different lowercase letters indicate significant differences (*p* < 0.05) among different nutrient-addition treatments under the same precipitation condition. (**a**) Effects of precipitation and nutrient deposition on the leaves biomass of *L. chinensis*. (**b**) Effects of precipitation and nutrient deposition on the stems biomass of *L. chinensis*. (**c**) Effects of precipitation and nutrient deposition on the sheaths biomass of *L. chinensis*. (**d**) Effects of precipitation and nutrient deposition on the aboveground biomass of *L. chinensis*.

Leaf area was not affected by precipitation, N deposition, or P deposition, and specific leaf area was affected only by nitrogen deposition (Table 1). Under average precipitation, N deposition and N and P co-deposition significantly increased the specific leaf area. Under increasing precipitation, the specific leaf area was the largest when N and P were co-deposited, but the difference was not significant when compared with the no-nutrient deposition treatment (Figure 3b). Regardless of how precipitation changed, N deposition, P deposition, and N and P co-deposition increased leaf thickness. When precipitation and P deposition increased at the same time, leaf thickness was the largest, but it did not reach the level of significance (Figure 3c). There were no significant differences in leaf dry matter content among treatments (Figure 3d).

#### 2.2.2. Leaf Photosynthesis-Related Traits

**Figure 4:**
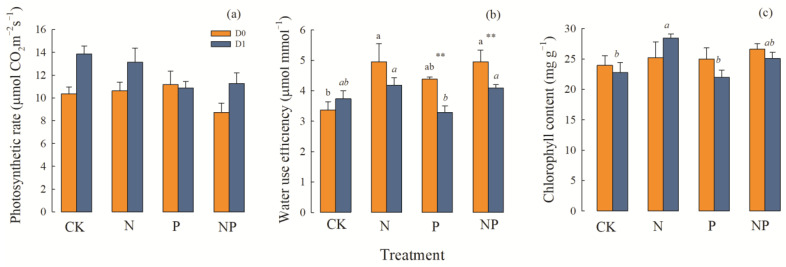
Effects of precipitation and nutrient deposition on the photosynthetic rate, water use efficiency, and chlorophyll content of *L. chinensis* (mean ± SE, *n* = 6). CK—control; N—simulated nitrogen deposition; P—simulated phosphorus deposition; NP—N and P co-deposition; D0—average precipitation; D1—increased precipitation. Different lowercase letters indicate significant differences (*p* < 0.05) among different nutrient-addition treatments under the same precipitation condition. The asterisk indicate significant differences (** *p* ≤ 0.01) among different precipitation condition under the same nutrient-addition treatments. (**a**) Effects of precipitation and nutrient deposition on the leaves biomass of *L. chinensis*. (**b**) Effects of precipitation and nutrient deposition on the stems biomass of *L. chinensis*. (**c**) Effects of precipitation and nutrient deposition on the sheaths biomass of *L. chinensis*. (**d**) Effects of precipitation and nutrient deposition on the aboveground biomass of *L. chinensis*.

The net photosynthetic rate (A) was significantly affected by precipitation and simulated P deposition (*p* < 0.01). The water use efficiency (WUE) was significantly affected by precipitation and N deposition, whereas the chlorophyll content was significantly affected only by N deposition (Table 1). Under average precipitation, nutrient deposition had no obvious effect on the photosynthetic rate, but when precipitation increased, N deposition and N and P co-deposition increased the net photosynthetic rate (Figure 4a). N deposition and N and P co-deposition increased WUE remarkably, attaining its largest value during N and P co-deposition under average precipitation conditions. However, when the precipitation increased, the WUE significantly decreased (*p* ≤ 0.01), and there was no significant difference in WUE among the nutrient deposition treatments (Figure 4b). In terms of chlorophyll content, the three simulated nutrient deposition combinations had no significant effect under average precipitation. Conversely, N deposition significantly increased the chlorophyll content under increasing precipitation, but the other nutrient deposition treatments had no significant effect on chlorophyll (Figure 4c).

### 2.3. Effects of Functional Traits on Aboveground Biomass

According to stepwise regression, with the average precipitation treatment, only WUE had significant effects on aboveground biomass (*p* ≤ 0.05), and the excluded variables were plant height, leaf area, specific leaf area, leaf thickness, leaf dry matter content, net photosynthetic rate, and chlorophyll content (Table 2). Under the condition of increasing precipitation, only plant height had significant effects on aboveground biomass (*p* ≤ 0.01), and the excluded variables were leaf area, specific leaf area, leaf thickness, leaf dry matter content, net photosynthetic rate, water use efficiency, and chlorophyll content (Table 2).

### 2.4. The Trade-Off Relationship between Plant Height, WUE, and Leaf Functional Traits

**Figure 5:**
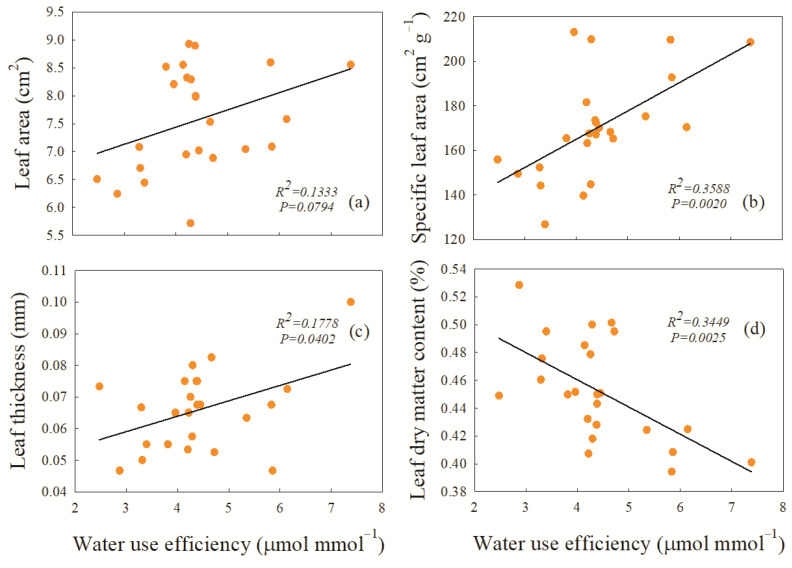
Correlation analysis between the water use efficiency and functional traits of *L. chinensis* under average precipitation conditions. The functional traits are (**a**) leaf area of *L. chinensis*; (**b**) specific leaf area of *L. chinensis*; (**c**) leaf thickness of *L. chinensis*; (**d**) leaf dry matter content of *L. chinensis*.

The correlation analysis between WUE and leaf functional traits under average precipitation showed that there was a significant positive correlation between WUE and specific leaf area (*p* ≤ 0.01) (Figure 5b) and a significant positive correlation between WUE and leaf thickness (*p* ≤ 0.05) (Figure 5c). The leaf area, specific leaf area, and leaf thickness increased with increasing WUE. However, there was a marked significant negative correlation between WUE and leaf dry matter content (*p* ≤ 0.01) (Figure 5d), i.e., there was a significant trade-off between them.

**Figure 6:**
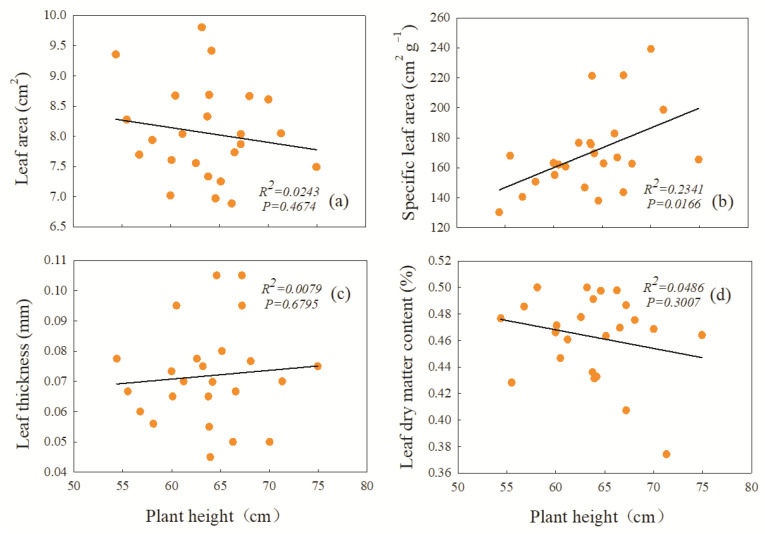
Correlation analysis between plant height and functional traits of *L. chinensis* under increasing precipitation conditions. The functional traits are (**a**) leaf area of *L. chinensis*; (**b**) specific leaf area of *L. chinensis*; (**c**) leaf thickness of *L. chinensis*; (**d**) leaf dry matter content of *L. chinensis*.

The correlation between plant height and leaf functional traits under increasing precipitation shows that there was a highly significant positive correlation between plant height and specific leaf area; that is, the taller the plant, the greater the specific leaf area (*p* ≤ 0.05) (Figure 6b). Nevertheless, plant height was negatively correlated with leaf area and leaf dry matter content, indicating that there was a significant trade-off between plant height and leaf area and leaf dry matter content. With the increase in *L. chinensis* plant height, the leaf functional traits of leaf area and leaf dry matter content showed a downward trend (Figure 6a,d).

## 3. Discussion

### 3.1. Response of Aboveground Biomass to Precipitation Changes and N and P Depositions

In terrestrial ecosystems, nitrogen (N) and phosphorus (P) are the most important limiting nutrients of plant productivity [16]. Additions of N and P can promote plant growth and increase plant biomass [17]. In arid and semi-arid regions, plant growth is limited not only by nutrients but also soil moisture [18]. The results of our experiment showed that under the main treatment of N and P, the aboveground biomass increased with the increase in precipitation, which indicates that a lack of moisture significantly restricts *L. chinensis* growth. This restriction is due to the fact that the absorption and transportation of nutrients by plants depend on water, and nutrients can be absorbed by plants only when those nutrients are dissolved in water [19]. Therefore, under the nutrient addition treatment, increasing precipitation would increase the nutrients dissolved in water, which is more conducive to promoting plant growth. We found that no matter how the precipitation changed, N deposition and N and P co-deposition both significantly increased the aboveground biomass, and P deposition had no significant effect on aboveground biomass, indicating that growth was limited mainly by N, while P deposition could promote the absorption of N.

### 3.2. Responses of Leaf Functional Traits to Precipitation Changes and N and P Depositions

Plant functional traits reflect the response and adaptation strategies of plants to environmental changes. Plants adapt to environmental heterogeneity by balancing different functional traits [20], especially leaf phenotypic plasticity [21]. Under drought conditions, the net photosynthetic rate of plants decreases, and the transpiration rate of plants decreases in response to reduced water loss [22]. In contrast, when precipitation increases, the net photosynthetic rate and transpiration rate of plants increase [23].

In the results of this experiment, WUE was increased under average precipitation, which may be due to the combined effect of net photosynthetic rate and transpiration rate on WUE. Previous studies have shown that when N is added, plants increase their light capture ability by increasing plant height. This enhances photosynthesis and provides them with a dominant position in growth and competition [24], which is accompanied by an improved plant net photosynthetic rate and WUE [25]. The simulated N deposition in this experiment significantly increased the plant height, which was consistent with previous studies. Chlorophyll is an important factor that determines the photosynthetic capacity of plant leaves, and it is closely related to the nitrogen content in the leaf [26]. Our results showed that N deposition significantly increased chlorophyll (a + b) content under the increasing precipitation treatment, which was consistent with previous studies [27]. In contrast, other simulated nutrient deposition treatments had no significant effect. This may be because P is not a key limiting nutrient in *L. chinensis* grassland [28].

Leaves are the main location for carbon fixation in plants. Leaf size affects light interception and the carbon acquisition ability of plants, which is closely related to photosynthesis [29,30], whereas the specific leaf area reflects the carbon acquisition strategy of plants [31], which is a key trait by which plants adapt to changes in the external environment [32]. Our results showed that the specific leaf area reached its largest values under simulated N deposition with average precipitation and under the co-deposition of N and P with increased precipitation. However, P addition had no significant effect on the leaf area, indicating that there was a significant coupling effect between N and P, and P addition might promote the uptake of N [33].

Under average precipitation, the biomass of *L. chinensis* had a highly significant positive correlation with the WUE of leaves, and the WUE had a mutually reinforcing relationship with leaf area, specific leaf area, and leaf thickness; however, there was a significant trade-off relationship between WUE and leaf dry matter content. As the precipitation increased, the biomass change was related mainly to the plant height traits. Thus, it was shown that *L. chinensis* adapted to the changes in the environmental conditions by balancing the relationship between plant height and leaf area and leaf dry matter traits. The mechanisms for this can be summarized as follows: when plants (*L. chinensis* was selected as the experimental material) are in water-limited conditions, nutrient deposition will increase WUE by appropriately increasing specific leaf area and leaf thickness to adapt to the relatively water-deficient environment; in contrast, when the water limitation was alleviated, nutrient deposition significantly increased plant height and specific leaf area, resulting in the enhancement of photosynthetic carbon capture ability. Under this condition, the relationship between plant height and dry matter content in leaves was balanced to avoid the possible lodging caused by the simultaneous increase in plant canopy and plant height, which was not conducive to the accumulation of total biomass.

## 4. Materials and Methods

### 4.1. Experimental Materials

In this experiment, *L. chinensis*, a mono-dominant perennial rhizome grass, widespread in the meadow steppe of the eastern semi-arid area of the Eurasian grassland belt, was selected as the experimental material. It has a strong clonal propagation ability and adaptability and has characteristics of cold resistance, drought resistance, and salt tolerance. The regrowth of the species occurs around mid–late April each year, and it often matures in late July and enters the post-fruit nutritional period in early August. At the beginning of October, the aboveground parts of plants wither and stop growing. The species is not only the main food source of local herbivores such as cattle and sheep but also plays an important role in soil and water conservation and ecological construction [15]. This study examined a native plant species that does not need any special licensing. This project was authorized by the Key Laboratory of Vegetation Ecology, Ministry of Education, Northeast Normal University.

### 4.2. Experimental Design

The mesocosm experiment corresponded to a three-factor factorial design with six replications. The factors included precipitation (average precipitation: 340 mm, increased precipitation: 440 mm), N deposition (N: 0, 10 g N ha^−1^ year^−1^), and P deposition (P: 0, 10 g P ha^−1^ year^−1^), with a total of eight treatments. The mesocosm is cylindrical with an inner diameter of 34.2 cm and a height of 43.5 cm, and it was filled with 50 kg of ground-surface soil (0–20 cm depth) collected from the natural habitat in the Songnen grassland area. Before the mesocosm was filled, the soil was sieved to remove the larger particle sizes (2 cm). After that, the soil was fully mixed and loaded into a pot to adjust the soil moisture content to an amount close to the field moisture capacity (15%). The soil bulk density of the 5–25 cm soil layer in the container was 1.61 g cm^−3^, and the total N, total P, available N, and available P in the soil were 0.7 ± 0.01 mg g^−1^, 0.012 ± 0.002 mg g^−1^, 2.4 ± 0.13 μg g^−1^, and 3.0 ± 0.46 μg g^−1^, respectively. Each mesocosm accommodated 30 plants. From May to October, N fertilizer (ammonium nitrate) was added to the soil successively in the form of pulse treatments alongside the rainfall treatments. P (superphosphate) was applied to the soil early in the months of May, June, July, August, September, and October in the same form. Soil collection and planting of the plant material were completed in 2016. After natural growth in 2017, plant materials were treated with additions of N and P and watered in 2018. Data collection was conducted at the peak of the growing season in July 2019.

In order to eliminate the influence of natural precipitation on the experiment, all experimental materials were arranged in greenhouses covered in plastic, and the precipitation was controlled via artificial watering. At the same time, in order to prevent any impact from the plastic covering on ambient temperature, the greenhouses were always left open around all sides. The precipitation data for 50 years near the Songnen Grassland Ecological Research Station of Northeast Normal University (from the weather station at the Songnen Grassland Ecological Research Station of Northeast Normal University) were statistically analyzed. The monthly average precipitation in various years was calculated, and the growing season average precipitation (D0: 340 mm) and precipitation increase (average precipitation + 30%: D1, 440 mm) were set from this. The precipitation frequency in the experiment was determined according to the average precipitation frequency during the growing seasons of the previous 50 years, and the precipitation frequency in the increasing precipitation treatment was the same as in the average precipitation condition.

### 4.3. Sampling and Determination Methods

Plant biomass, which includes leaf biomass, stem biomass, and sheath biomass, was determined as follows. First, 10 healthy and non-damaged individual plants in each mesocosm were sampled to determine the plant height. Then, the stems, leaves, and sheaths of *L. chinensis* were quickly separated and weighed after being dried at 65 °C for 48 h. Leaf functional traits including leaf area (LA), specific leaf area (SLA), and leaf thickness (LT) were measured. In each mesocosm, two fully expanded healthy leaves were selected, and a leaf area meter (AM350) was used to determine the leaf area; then, the leaves were dried to a constant weight to determine their mass (LM). The specific leaf area was calculated by using the formula SLA = LA/LM. Leaf thickness (LT, mm) was measured with an absolute zero electronic digital display vernier caliper (accuracy: 0.01 mm). Then, the samples were placed into a test tube and extracted with 10 mL of a 1:1 mixture of 80% acetone and anhydrous ethanol.

Photosynthetic parameters including net photosynthetic rate (A) and water use efficiency (WUE) of the first fully expanded leaf at the top of the plant were measured with a photosynthetic apparatus (CIRAS-3, PP Systems, Amesbury, MA, USA). In order to avoid the influence of natural light intensity and other disturbances, the light intensity and CO_2_ concentration were controlled by the red–blue–white simulated light source and CO_2_ injection system. The A, stomatal conductance, and intercellular CO_2_ concentration were measured at 1000 μmol^−2^ S^−1^ light intensity and 380 μmol mol^−1^ CO_2_ concentration. Finally, the WUE was obtained by calculating the ratio of the net photosynthetic rate to the stomatal conductance.

After that, 0.1 g of fresh leaf samples were collected to determine the photosynthetic pigments, including chlorophyll a and chlorophyll b. The leaf samples were cut into small pieces of uniform size, placed into a test tube, extracted with 10 mL of a 1:1 mixture of 80% acetone and anhydrous ethanol 1:1, and then placed in dark conditions to soak and extract until the leaf samples were fully white. Then, the absorbance values of the samples were measured at 663 nm and 645 nm with a UV-754 spectrophotometer. The photosynthetic pigment content was calculated according to the following formulae: Ca = 9.784 × A663 − 0.990 × A645, Cb = 21.426 × A645 − 4.65 × A663.

### 4.4. Data Analysis

Data analysis was performed using SPSS 22.0 (IBM SPSS Inc., Chicago, IL, USA). An assumption test was performed on all data before data analysis. Under the condition that the data were in line with the normality test and homogeneity of variance, the effects of precipitation, simulated N deposition, P deposition, and their interactions on the functional traits and aboveground biomass were analyzed by using a two-way ANOVA under the general linear model. The relationship between biomass and functional traits was obtained via a stepwise regression analysis. Because there was no significant interaction among precipitation, N deposition, and P deposition for various functional traits, this study was divided into two groups of average precipitation and increased precipitation for stepwise regression analysis so as to screen out plant functional traits that had significant effects on aboveground biomass. The trade-offs of key traits were analyzed via Pearson correlation analysis (double-tail test, α = 0.05). According to Tukey-b variance test analysis, the mean value of each group marked with different letters represented significant difference (*p* ≤ 0.05).

## 5. Conclusions

Our results showed the following: (1) the aboveground total biomass and biomass of each organ (leaf, stem, and sheath) increased with the increase in precipitation; (2) N addition and N and P co-addition promoted biomass production, whereas P addition alone had little effect on biomass accumulation, regardless of precipitation treatments; (3) WUE is the key trait that determines *L. chinensis* biomass under insufficient precipitation, but when water availability increases, plant height is the crucial trait for determining biomass production. Indeed, against the backdrop of nutrient deposition, high or low water availability has a decisive impact on the importance of various traits. In summary, under N and P deposition conditions, the shift in key photosynthetic carbon capture traits caused by precipitation is the key ecological driving mechanism for the biomass production of typical dominant plant species in semi-arid grasslands.

## Figures and Tables

**Table 1:** Variance analysis of the effects of precipitation, N and P deposition, and their interaction on the biomass and functional traits of *L. chinensis*.

Factor	Precipitation	N	P	Precipitation × N	Precipitation × P	N × P	Precipitation × N × P
	F	F	F	F	F	F	F
Leaf biomass	14.155 **	63.829 **	0.75	0.412	0.002	0.002	8.581 **
Stem biomass	8.485 **	90.028 **	0.333	0.263	0.683	1.785	4.875 *
Sheath biomass	12.116 **	71.361 **	0.108	0.298	0.025	0.234	8.243 **
Aboveground biomass	13.490 **	77.924 **	0.138	0.384	0.044	0.206	8.304 **
Plant height	9.047 **	31.563 **	5.772 *	0.901	5.679 *	6.200 *	1.210
Leaf area	3.794	0.941	0.123	0.714	2.056	0.141	1.347
Specific leaf area	0.003	14.076 **	1.242	0.217	0.428	0.469	0.003
Leaf thickness	1.972	0.041	2.622	0.419	0.022	2.201	0.032
Leaf dry matter content	1.290	5.380 *	0.786	3.047	0.389	0.101	0.001
Net photosynthetic rate	11.050 **	0.982	5.741 *	0.572	2.350	0.409	2.388
Water use efficiency	7.279 **	15.132 **	0.295	1.089	3.138	0.536	2.478
Chlorophyll a + b	0.313	7.030 *	0.156	1.822	2.280	0.248	0.424

Note: ** *p* ≤ 0.01; * *p* ≤ 0.05.

**Table 2:** Stepwise regression analysis of the effects of functional traits of *L. chinensis* on aboveground biomass under control and increased precipitation treatments.

	Responding Variable	Predictive Variable	β	T	R2	F
Control	Aboveground biomass	Water use efficiency	0.445	2.334 *	0.198	5.447 *
Precipitation		Plant height	0.550	3.086 **	0.302	9.524 **

Note: ** *p* ≤ 0.01; * *p* ≤ 0.05.

## Data Availability

Research data are not shared.
